# CADASIL‐like leukodystrophy and symptomatic cerebral infarction in myotonic dystrophy type 1

**DOI:** 10.1111/cns.13908

**Published:** 2022-07-03

**Authors:** Bin Liu, Chun‐Lin Yang, Xiao‐Li Li, Min Zhang, Yan‐Bin Li, Rui‐Sheng Duan

**Affiliations:** ^1^ Department of Neurology The First Affiliated Hospital of Shandong First Medical University & Shandong Provincial Qianfoshan Hospital Jinan China; ^2^ Shandong Institute of Neuroimmunology Jinan China

**Keywords:** CADASL, ischemic stroke, MRI, myotonic dystrophy type 1

Dear Editor,

Myotonic dystrophy type 1 (DM1) is an autosomal dominant multisystemic disorder that causes muscle weakness, atrophy, and myotonia. It is well known that DM1 is associated with white matter lesions in the brain. However, the characteristic magnetic resonance imaging (MRI) patterns in DM1 still have some weakness. Anterior temporal lobe hyperintensities and white matter hyperintensities are characteristics of cerebral autosomal dominant arteriopathy with subcortical infarcts and leukoencephalopathy (CADASIL).^1^ Because similar findings detected by brain MRI have been described in DM1, the brain MRI in these two diseases is often misinterpreted.

The patient was a 52‐year‐old woman presented with a 10‐year history of progressive limb weakness. Three months ago, the patient presented with sudden onset of left‐sided weakness. She was diagnosed with acute ischemic stroke at a local hospital. The patient had no medical history of diabetes, hypertension, metabolic, toxic, or any neurological diseases. Neurological examination revealed alopecia, neck flexion weakness, mild bilateral facial weakness, interosseous muscle atrophy in both hands, left limb weakness (3/5), and right limb weakness (4/5). No abnormalities in the cranial nerves and sensory system were noted.

In laboratory tests, serum triglycerides (2.77 mmoL/L), serum lactate dehydrogenase (LDH 310 U/L), alanine transaminase (ALT 207.0 U/L), and glutamic oxalacetic transaminase (AST 93.0 U/L) were slightly elevated. Creatine kinase was 45 U/L. The test was also negative for anti‐neutrophils cytoplasmic antibodies, autoantibodies, and female tumor markers. Needle electromyography showed myotonic discharges in proximal and distal muscles. Ophthalmologic examination showed bilateral cataracts. Electrocardiography and 24‐h electrocardiogram showed no abnormalities.

An MRI of the brain showed CADASIL‐like leukodystrophy. Multifocal T2 and FLAIR hyperintensities were mainly located in the white matter of the frontal, parietal, and anteromedial temporal lobes (Figure 1A–D). T1‐weighted and T2‐weighted images showed cerebral infarction in right cerebral peduncle 3 months ago (E and F). MRA showed cerebral arteriosclerosis (Figure 1G). Axial susceptibility weight‐imaging (SWI) images showed the absence of microbleeds (Figure 1H). T2‐weighted image showed enlarged Virchow‐Robin spaces in the centrum semiovale (Figure 1I). Genetic testing showed an over 100 expansion of CTG‐repeats in the DMPK gene on chromosome 19q13.3, confirming the diagnosis of myotonic dystrophy.

**FIGURE 1:**
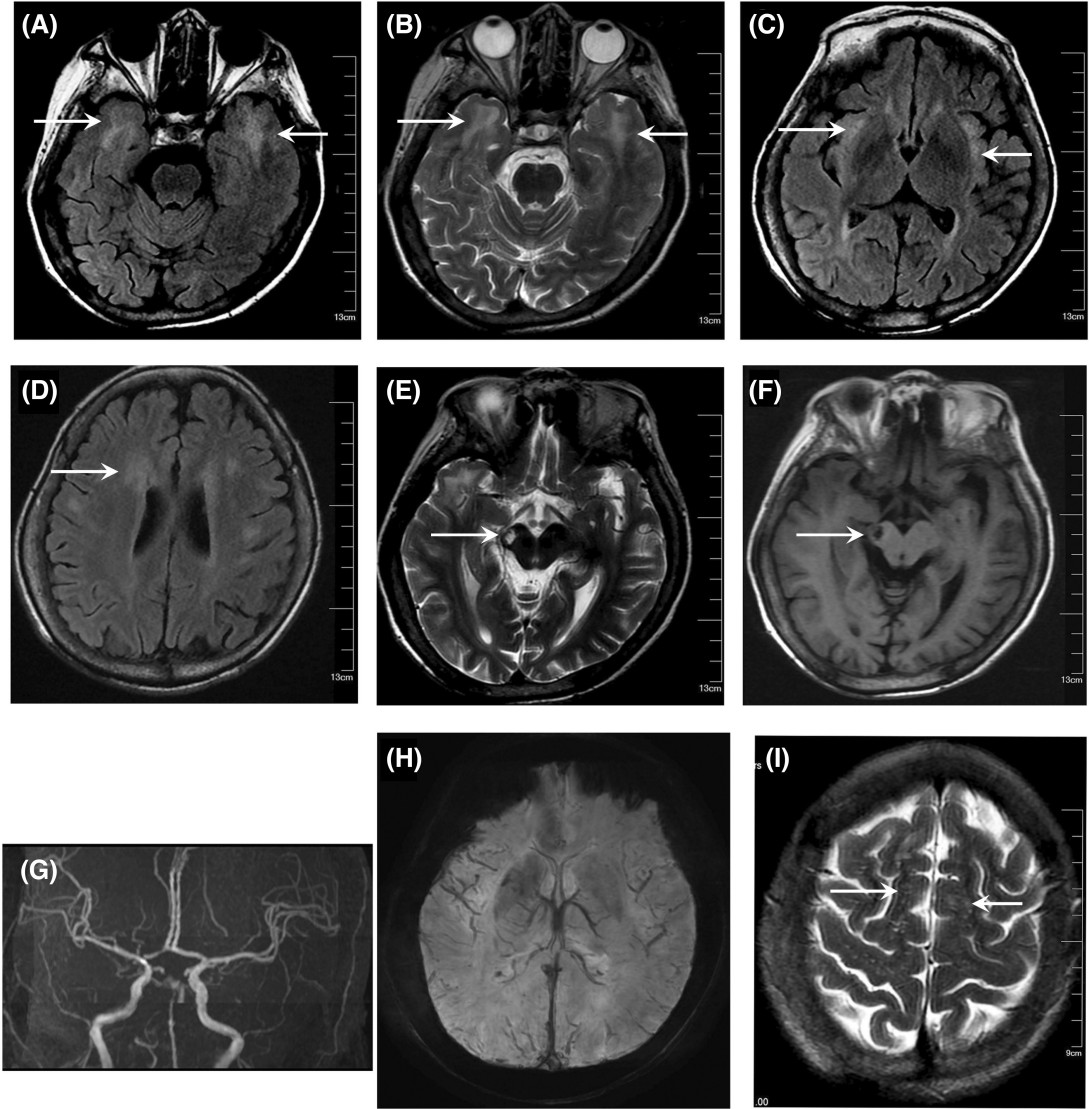
MRI images of this patient. Axial FLAIR (A) and T2‐weighted (B) images at the same level show white matter lesions in the temporal lobes. Axial FLAIR‐weighted images (C and D) show confluent white matter lesions in all cerebral lobes and signs of brain atrophy. Cerebral infarction in right cerebral peduncle 3 months ago (E and F). MRA show basilar artery arteriosclerosis (G). Axial susceptibility weight‐imaging (SWI) images showing the absence of microbleeds (H). Axial T2‐weighted image shows prominent Virchow‐Robin spaces in the centrum semiovale (I)

In this report, the patient presented with myotonia, muscle wasting, alopecia, and cataracts characterizing DM1. Subcortical hyperintensity of the white matter on T2/FLAIR brain MRI, with frequent involvement of the anterior temporal lobe, are common findings in DM1.^2^ Gray and white matter atrophy and dilated Virchow‐Robin spaces are also commonly described in DM1 patients.^3^ Involvement of the anterior part of the temporal lobes is highly suggestive for CADASIL. However, anterior temporal lobe involvement in DM1 is not infrequent compared with CADASIL. Hence, the brain MRI in these two diseases is often confused. This patient had lacunar infarction and MRI showed CADASIL‐like hyperintensities. Thus, she was initially misdiagnosed as CADASIL. However, MRI exhibited specific features that could aid in discriminating DM1 from CADASIL. Incident lacunes, cerebral microbleeds, enlarged perivascular space, and brain atrophy are often observed in CADASIL. Among these, brain atrophy and the number of incident lacunes are sensitive MRI markers that could predict clinical worsening in CADASIL.^4^ Following a study by Kim et al. in 2018, frontal and parieto‐occipital lobes, external capsule, and basal ganglia were involved to a lesser extent in DM1 than in CADASIL. Cerebral microbleeds were not observed in any of the MRI images of patients with DM1, whereas 69% of those with CADASIL had microbleeds.^5^ Brain MRI characteristics in patients with DM1 or CADASIL are presented in Table 1. The prevalence of stroke was also less common in DM1 than in CADASIL.^5^ Ischemic stroke in patients with myotonic dystrophy type 1 is mainly associated with atrial fibrillation and arteriosclerosis.^6^ Hypertriglyceridemia, hypertension, and hyperglycemia were very common in DM1 patients,^7^ and severe arteriosclerosis was reported in myotonic dystrophy.^8^ This patient has a stroke syndrome and neuroimaging findings consistent with arteriosclerosis. However, studies about arteriosclerosis and cerebral infarction in DM1 patients have not been fully conducted. Even though the MRI in patients with DM1 and CADASIL revealed similar anterior temporal lobe T2 hyperintensity, the pathologic mechanisms underlying the two diseases are distinct. DM1 is caused by an unstable expanded CTG repeats in the noncoding regions of DMPK gene.^9^ CADASIL is usually caused by NOTCH3 gene mutations, which provide another biomarker for the differentiation between DM1 and CADASIL.^10^

**TABLE 1:** Brain MRI characteristics in patients with DM1 or CADASIL

	DM1	CADASIL
White matter changes		
Total	Common (over 60%)	Very common (over 90%)
Frontal	Common (over 30%)	Very common (over 90%)
Parieto‐occipital	Common (over 50%)	Very common (over 90%)
Anterior temporal	Common (approximately one‐third)	Common (over 50%)
External capsule	Rare (about 10%)	Very common (over 80%)
Basal ganglia	Very rare	Common (over 60%)
Stroke	Rare (about 10%)	Very common (over 70%)
Presence of microbleeds	Very rare	Common (over 60%)

Abbreviations: CADASIL, cerebral autosomal dominant arteriopathy with subcortical infarcts and leukoencephalopathy; DM1, myotonic dystrophy type 1.

## CONFLICTS OF INTEREST

The authors have declared that no competing interest exists.

## INFORMED CONSENT

We have obtained the patient's permission and informed consent for the publishing of her information and images.

## Data Availability

Data available on request from the authors.
